# Multimorbidity and complex multimorbidity, their prevalence, and associated factors on a remote island in Japan: a cross-sectional study

**DOI:** 10.1186/s12875-022-01860-2

**Published:** 2022-10-03

**Authors:** Yoshifumi Sugiyama, Rieko Mutai, Takuya Aoki, Masato Matsushima

**Affiliations:** 1 Division of Clinical Epidemiology, Research Center for Medical Sciences, The Jikei University School of Medicine, 3-25-8, Nishishimbashi, Minato-ku, Tokyo 105-8461 Japan; 2 Tarama Clinic, Okinawa Miyako Hospital, 162-3, Shiokawa, Tarama, Miyako-gun, Okinawa, 906-0601 Japan; 3 Division of Community Health and Primary Care, Center for Medical Education, The Jikei University School of Medicine, 3-25-8, Nishishimbashi, Minato-ku, Tokyo 105-8461 Japan; 4 Department of Adult Nursing, The Jikei University School of Nursing, 8-3-1, Kokuryocho, Chofu, Tokyo 182-8570 Japan

**Keywords:** Multimorbidity, Complex multimorbidity, Associated factor, Remote island

## Abstract

**Background:**

Evidence is still limited on the prevalence of multimorbidity and complex multimorbidity in Japan, as well as their associated factors. Additionally, no studies regarding multimorbidity and complex multimorbidity in Japan have used patient data obtained directly from medical records. The primary objective was to clarify the prevalence of multimorbidity and complex multimorbidity using patient data obtained directly from medical records. The secondary objective was to elucidate factors associated with multimorbidity and complex multimorbidity.

**Methods:**

We conducted a cross-sectional study at Tarama Clinic on Tarama Island, a remote island in Okinawa, Japan. Among patients who visited Tarama Clinic from April 1 to June 30, 2018, those who were ≥ 20 years of age, lived on Tarama Island, and had capacity to give consent were eligible for study inclusion. We collected the following data using medical records, medical expense receipts, and self-administered questionnaires: multimorbidity and complex multimorbidity, participants’ characteristics, and potentially associated factors. Multimorbidity was defined as having ≥ 2 among 17 chronic health conditions, in accordance with previous studies conducted in Japan, and having ≥ 2 among 16 chapters of the International Classification of Primary Care, 2nd edition (ICPC-2). Complex multimorbidity was defined as having ≥ 3 among 16 chapters of the ICPC-2.

**Results:**

We included 355 study participants. Descriptive analysis showed multimorbidity prevalence measured using the 17 chronic health conditions in this area was 30.0% (age ≥ 20 years) and 57.5% (age ≥ 65 years). Multimorbidity prevalence measured using the ICPC-2 was 32.9% (age ≥ 20 years) and 60.3% (age ≥ 65 years). We also found a 20.9% (age ≥ 20 years) and 45.1% (age ≥ 65 years) prevalence of complex multimorbidity, respectively. Poisson regression with robust error variance showed that age ≥ 60 years was associated with multimorbidity. Age ≥ 60 years (adjusted prevalence ratio [aPR] 1.887 and 1.831 for ages 60–79 and ≥ 80 years, respectively) and being unemployed (aPR 1.251) were associated with complex multimorbidity. However, having hazardous drinking or more was inversely associated with complex multimorbidity (aPR 0.745).

**Conclusions:**

The population-based prevalence of multimorbidity and its upward trend with increasing age on a remote island in Japan was consistent with previous reports in the country. Multimorbidity was associated with age 60 years or older, and complex multimorbidity was associated with age 60 years or older, being unemployed, and not having hazardous drinking or more. Our study findings indicated a possible association between the coexistence of diseases and social determinants of health (SDH) in Japan. To improve care for patients with multimorbidity and complex multimorbidity, more research that takes SDH into account is warranted, and evidence-based policymaking is essential for Japan. The present study can provide a foundation for accumulating such evidence.

## Background

Multimorbidity, which is generally defined as the coexistence of two or more diseases or conditions in one individual [1], is increasing owing to several factors, including aging of the population [2, 3]. Multimorbidity is associated with a higher risk of worse health outcomes, such as increased mortality [4] or decreased quality of life [5]. Multimorbidity also leads to an increased burden of treatment, such as higher consultation rates, less continuity of care [6], or polypharmacy [7]. A meta-analysis of observational studies reported an overall global prevalence of multimorbidity of 33.1% [3]. Determinants of multimorbidity include sex (women) and lower socioeconomic status as well as age [8].

Multimorbidity is an increasingly important topic in Japan. The population aged 65 years or more is growing owing to increasing life expectancy and declining birth rates [9]; thus, multimorbidity has been drawing greater attention as a public health issue in Japan and worldwide. In terms of population aging, Japan is at the forefront of the trend. As of October, 2020, Japan’s proportion of the population aged 65 years or more reached 28.8%, the highest in the world [10]. Accordingly, multimorbidity is becoming an unavoidable problem that Japan must address sooner than other countries.

To date, a very limited number of articles have been published on multimorbidity in Japan [11–14]. In addition to multimorbidity, during the past decade, the idea of “complex multimorbidity,” which is defined as the co-occurrence of three or more chronic conditions affecting three or more different body systems, has been proposed [15]. Complex multimorbidity differentiates patients whose treatment and care are more complex because chronic conditions in different body systems are likely to require multiple types of treatment and care [15]. As with multimorbidity, a very limited number of articles have been published on complex multimorbidity in Japan [16, 17].

It is difficult to investigate multimorbidity and complex multimorbidity precisely in Japan. The health care system allows Japanese patients to select and visit any clinics or hospitals that they wish, which is called a “free–access” system [18]. Thus, patients’ data, including information regarding diseases, is dispersed among medical institutions, making it difficult to obtain the data collectively. Therefore, all past studies regarding multimorbidity and complex multimorbidity in Japan have not obtained patients’ data directly from the medical records but rather from a database containing self-reported data [11, 12, 14, 16, 17] or from a large-scale, anonymized medical claims database [13]. Differing methods to assess multimorbidity influence the prevalence estimations [2]. For example, in studies that use self-reported data, response or recall bias cannot be completely avoided [3]. Additionally, the prevalence of some diseases, including peptic ulcer disease, cancer, cerebrovascular disease, or coronary heart disease, are probably overestimated when using a medical claims database [13].

Accordingly, evidence is still limited on the prevalence of multimorbidity and complex multimorbidity in Japan, as well as their associated factors. Additionally, no studies regarding multimorbidity and complex multimorbidity in Japan have used patient data obtained directly from medical records. When there are a limited number of medical institutions in one area and that area is geographically isolated from the rest of the country (e.g., by the ocean), residents are expected to visit the medical institutions in the area. This unique situation limits scattering of patients’ data across medical institutions and enables us to obtain data collectively. Therefore, we conducted the present study on Tarama Island, a remote island in Okinawa, Japan. The primary objective of this study was to clarify the prevalence of multimorbidity and complex multimorbidity using patient data obtained directly from the medical records. The secondary objective was to elucidate factors associated with multimorbidity and complex multimorbidity.

## Methods

In this study, we analyzed the data collected using medical records, medical expense receipts, and self-administered questionnaires in a previous study on the association between alcohol consumption/alcohol use disorders and patient complexity [19].

### Study design

This was a cross-sectional study. This study is reported in accordance with the Strengthening the Reporting of Observational Studies in Epidemiology (STROBE) guidelines [20].

### Setting

We conducted this study at Tarama Clinic, Okinawa Miyako Hospital [21] on Tarama Island, a remote island in Okinawa, Japan. Tarama Island is a main part of Tarama Village and is located between Miyako Island and Ishigaki Island, approximately 67 km west of Miyako Island and approximately 35 km northeast of Ishigaki Island [22]. Travel to Tarama Island from Miyako Island by airline or ferry takes 25 min and 125 min, respectively [23, 24]. Miyako Island is located approximately 300 km southwest of the main island of Okinawa [25], which is the westernmost prefecture in Japan. In 2015, Tarama Village had a population of 1194 people (555 women and 639 men) [26]. In the same year, the proportion of the population aged 65 years or older was 26.4%, which was nearly the same as the national average of 26.6% [27]. A large proportion (41.6%) of the population was engaged in primary industries (agriculture, forestry, and fishery) [28, 29], compared with the national average (3.8%) [30] Specifically, raising beef cattle and growing sugar cane, vegetables, and tobacco are the leading industries [22]. The average income was 1,765,000 Japanese yen [JPY]/person [31], which was lower than the national average of 3,203,000 JPY/person in 2015 [32].

With four staff (a physician, a nurse, a nurse assistant, and a clerk), Tarama Clinic is the only medical institution other than a dental clinic in Tarama Village. The clinic provides general outpatient and around-the-clock emergency services [33]. When requiring specialized medical services or hospitalization, patients are transferred to a medical institution located off Tarama Island.

Most people on Tarama Island are expected to visit Tarama Clinic because of geographical constraints, which prevent people from willingly attending other medical institutions located off Tarama Island. Thus, this unique situation limits the dispersal of patients’ data across medical institutions and enabled us to conduct a population-based study, even in Japan.

### Participants

Among patients who visited Tarama Clinic from April 1 to June 30, 2018, those who were ≥ 20 years of age, lived on Tarama Island, and had capacity to give consent were eligible for study inclusion. We excluded patients who refused to participate in our previous study or whose participation was judged to have an unfavorable influence on patient–physician relationships. During data collection in our previous study, we assessed patient complexity using the Patient Centered Assessment Method (PCAM), which is a tool for assessing patient complexity [19]. The PCAM includes questions regarding personal issues, such as financial insecurity. Assessing patient complexity may be psychologically invasive and could impair patient–physician relationships. Considering the medical context of Tarama Island, patients have no choices regarding medical institutions other than Tarama Clinic. If a patient–physician relationship was impaired, the patient would likely drop out of any treatment being received. Therefore, we paid careful attention to this issue and excluded patients whose participation we judged could have an unfavorable influence on the patient–physician relationship. We also did not enroll any patients when the principal investigator was out of the office and could not obtain informed consent, or when a large number of patients were waiting to see the physician (the principal investigator) and obtaining informed consent would have interfered with outpatient practice. After the principal investigator fully informed potential participants about our previous study, those who agreed to participate provided their written informed consent. Additionally, information regarding the conduct of the present study was disclosed to all potential participants. They were also provided the opportunity to decline participation in the study.

### Data collection

We collected the following data between April 1, 2018 and March 31, 2019.

#### Multimorbidity and complex multimorbidity

We obtained information regarding diseases from patients’ medical records.

First, in accordance with previous studies conducted by Aoki et al. in Japan [11, 12], the following 17 chronic health conditions were selected: hypertension, diabetes, dyslipidemia, stroke, cardiac diseases, chronic respiratory diseases, digestive diseases, kidney diseases, urologic diseases, arthritis/rheumatism, lumbar diseases, neurologic diseases, mental disorders, endocrine diseases, malignancy, vision abnormalities, and skin diseases. Multimorbidity was defined as having ≥ 2 among the 17 chronic health conditions.

Second, to assess multimorbidity and complex multimorbidity based on body systems, the following 16 chapters of the International Classification of Primary Care, 2nd edition (ICPC-2) were used: general and unspecified; blood, blood forming organs and immune mechanism; digestive; eye; ear; cardiovascular; musculoskeletal; neurological; psychological; respiratory; skin; endocrine/metabolic and nutritional; urological; pregnancy, childbearing, family planning; genital (female genital and male genital); and social problems [34]. The ICPC-2 female and male genital system chapters were combined because they included the same body systems [15]. Multimorbidity and complex multimorbidity were defined as having ≥ 2 and ≥ 3 among the 16 chapters of the ICPC-2, respectively.

#### Participants’ characteristics and potentially associated factors

We obtained information regarding age and sex (“female” or “male”) from the medical records. For statistical analysis, we divided age into the following three categories: 20–59 years, 60–79 years, and 80 years or older. We obtained information regarding the receipt of public assistance under the welfare system (“not receiving” or “receiving”) from the medical expense receipts. We also obtained marital status (“married,” “single,” or “divorced or widowed”), living situation (“not living alone” or “living alone”), education (“elementary school or junior high school,” “high school or junior high school under the old system,” or “vocational school, junior college, technical school, university, college, graduate school, or other”), work status (“working” or “not working”), physical activity (“exercising" or “not exercising”), smoking status (“ex-smoker or never smoker” “current smoker”) in self-administered questionnaires. In terms of alcohol intake, we obtained scores on the Alcohol Use Disorders Identification Test (AUDIT) [35] using self-administered questionnaires. The AUDIT is a 10-item screening tool to identify hazardous drinking, harmful drinking, and alcohol dependence. Each item is scored 0, 1, 2, 3, or 4; or 0, 2, or 4; thus, AUDIT scores range from 0 to 40 points. In accordance with guidelines and an article regarding cutoff values, AUDIT scores were divided into the following two categories: < 12 points (“not having hazardous drinking or more”) and ≥ 12 points (“having hazardous drinking or more”) [35–37]. A nurse supported patients in completing the questionnaires, if desired or needed.

### Statistical analysis

We performed descriptive analysis to clarify the prevalence of multimorbidity and complex multimorbidity. Calculating the prevalence of multimorbidity, we used 17 chronic health conditions and 16 ICPC-2 chapters. In calculating the prevalence of complex multimorbidity, we used 16 ICPC-2 chapters. We used the population aged ≥ 20 years (916 people) and ≥ 65 years (315 people), obtained using publicly available data [27] to calculate population-based prevalence, assuming that all patients with multimorbidity and complex multimorbidity on Tarama Island visited Tarama Clinic during the inclusion period. We also divided the population into age groups (20–29 years, 69 people; 30–39 years, 107 people; 40–49 years, 139 people; 50–59 years, 191 people; 60–69 years, 169 people; 70–79 years, 137 people; and 80 years or older, 104 people) to calculate the population-based prevalence by age group. Descriptive analysis was expressed as mean (standard deviation [SD]) for continuous variables and number (%) for categorical variables.

We also performed Poisson regression with robust error variance to elucidate factors associated with multimorbidity and complex multimorbidity, using the following variables: age, sex, marital status, living situation, education, work status, public assistance, physical activity, smoking status, and alcohol intake. In this analysis, the 16 ICPC-2 chapters were used to categorize multimorbidity and complex multimorbidity. *P*-values < 0.05 were considered statistically significant.

We performed descriptive analysis and Poisson regression with robust error variance using STATA/MP version 17.0 [38].

## Results

**Fig. 1:**
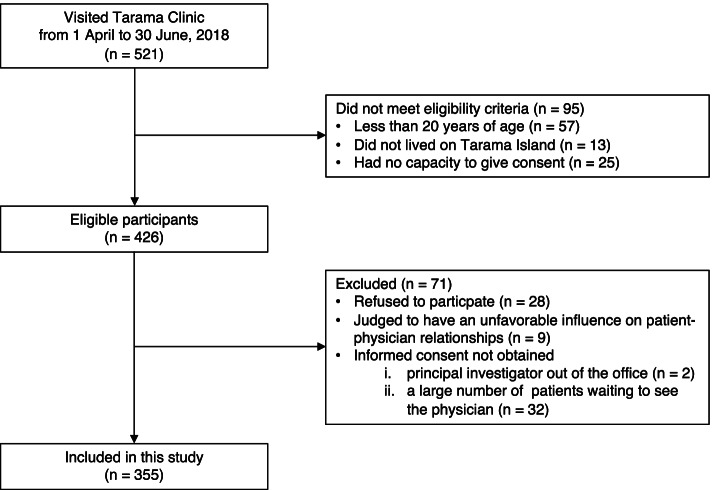
Inclusion and exclusion of study participants. This figure is modified from Sugiyama et al. [19]

During the inclusion period, 521 patients visited Tarama Clinic. Among them, 95 did not meet the eligibility criteria: 57 were < 20 years of age, 13 did not live on Tarama island, and 25 had no capacity to give consent. Among 426 eligible participants, 71 were excluded: 28 refused to participate in our previous study, participation was judged to have an unfavorable influence on patient–physician relationships in 9, informed consent could not be obtained from 2 because the principal investigator was out of the office, and informed consent could not be obtained from 32 because a large number of patients were waiting to see the physician and obtaining informed consent would have interfered with outpatient practice. As a result, 355 study participants were included in this study (Fig. 1).

The characteristics of the 355 study participants included in this study are shown in Table 1. The mean (SD) age was 66.4 (13.6) years.

**Table 1:** Characteristics of study participants

Total number of participants = 355		
	n	(%)
Age group		
20–59 years	108	30.4
60–79 years	183	51.6
80 years or older	64	18.0
Sex		
Female	163	45.9
Male	192	54.1
Marital status		
Married	246	69.3
Single	28	7.9
Divorced or widowed	81	22.8
Living situation		
Not living alone	289	81.4
Living alone	66	18.6
Education		
Elementary school or junior high school	187	52.7
High school or junior high school under the old system	115	32.4
Vocational school, junior college, technical school, university, college, graduate school, or other	53	14.9
Work status		
Working	307	86.5
Not working	48	13.5
Public assistance		
Not receiving	345	97.2
Receiving	10	2.8
Physical activity		
Exercising	53	14.9
Not exercising	302	85.1
Smoking status		
Ex-smoker or never smoker	305	85.9
Current smoker	50	14.1
Alcohol intake		
Not having hazardous drinking or more	244	68.7
Having hazardous drinking or more	111	31.3

This table is modified from Sugiyama et al. [19]

### Prevalence of multimorbidity and complex multimorbidity

The number and proportion of patients who had at least one disease in a category of the 17 chronic health conditions and a category of the 16 ICPC-2 chapters are shown in Table 2. The number of morbidities is also shown in Table 2.

**Table 2:** Patients with ≥ 1 disease in a category of the 17 chronic health conditions and the 16 ICPC-2 chapters, and morbidities

Total number of participants = 355					
Chronic health conditions			ICPC-2 chapters		
	n﻿	(%)		n	(%)
Hypertension	272	76.6	Cardiovascular	281	79.2
Dyslipidemia	188	53.0	Endocrine/metabolic and nutritional	248	69.9
Arthritis/rheumatism	88	24.8	Musculoskeletal	115	32.4
Lumbar diseases	88	24.8	Digestive	75	21.1
Diabetes	83	23.4	Neurological	73	20.6
Digestive diseases	69	19.4	Psychological	43	12.1
Cardiac diseases	51	14.4	Eye	35	9.9
Stroke	44	12.4	Respiratory	28	7.9
Vision abnormalities	31	8.7	Urological	25	7.0
Mental disorders	30	8.5	Genital (female genital and male genital)	25	7.0
Neurologic diseases	25	7.0	Skin	18	5.1
Chronic respiratory diseases	23	6.5	Ear	16	4.5
Kidney diseases	23	6.5	Blood, blood forming organs, and immune mechanism	10	2.8
Urologic diseases	18	5.1	General and unspecified	1	0.3
Skin diseases	17	4.8	Pregnancy, childbearing, family planning	0	0.0
Malignancy	11	3.1	Social problems	0	0.0
Endocrine diseases	4	1.1			
Number of morbidities			Number of morbidities		
0	10	2.8	0	3	0.9
1	70	19.7	1	51	14.4
2	72	20.3	2	110	31.0
≥ 3	203	57.2	≥ 3	191	53.8

*ICPC-2* International classification of primary care, 2nd edition

According to the 17 chronic health conditions, 275 patients aged ≥ 20 years and 181 patients aged ≥ 65 years had multimorbidity, resulting in 30.0% (275/916) and 57.5% (181/315) multimorbidity prevalence in the study area. By age group, we found 0.0% (20–29 years), 0.0% (30–39 years), 11.5% (40–49 years), 22.5% (50–59 years), 40.8% (60–69 years), 64.2% (70–79 years), and 56.7% (80 years or older) prevalence of multimorbidity.

According to the 16 ICPC-2 chapters, 301 patients aged ≥ 20 years and 190 patients aged ≥ 65 years had multimorbidity, resulting in 32.9% (301/916) and 60.3% (190/315) multimorbidity prevalence in this area. By age group, we found 0.0% (20–29 years), 2.8% (30–39 years), 12.9% (40–49 years), 28.8% (50–59 years), 43.2% (60–69 years), 65.7% (70–79 years), and 59.6% (80 years or older) prevalence of multimorbidity. Additionally, 191 patients aged ≥ 20 years and 142 patients aged ≥ 65 years had complex multimorbidity, which led to a 20.9% (191/916) and 45.1% (142/315) prevalence of complex multimorbidity in the study area.

### Factors associated with multimorbidity and complex multimorbidity

The results of Poisson regression with robust error variance, with multimorbidity as an objective variable, are shown Table 3. Considering the results regarding multimorbidity and referring to the appropriate number of events per variable of 10 or more in logistic regression analysis [39], we used three covariates, including age and sex, to perform the analyses. Other than age 60 years or older, no factors were found to be associated with multimorbidity.

**Table 3:** Poisson regression with robust error variance, with multimorbidity as an objective variable, and adjusted prevalence ratios

	**Model 1**			**Model 2**			**Model 3**			**Model 4**		
	**aPR**	**P-value**	**95% CI**	**aPR**	**P-value**	**95% CI**	**aPR**	**P-value**	**95% CI**	**aPR**	**P-value**	**95% CI**
Age												
20–59 years	Ref	–	–	Ref	–	–	Ref	–	–	Ref	–	–
60–79 years	1.253	0.001	1.097–1.431	1.273	< 0.001	1.114–1.453	1.243	0.002	1.083–1.426	1.265	0.001	1.107–1.446
80 years or older	1.346	< 0.001	1.167–1.554	1.388	< 0.001	1.208–1.595	1.342	< 0.001	1.150–1.566	1.375	< 0.001	1.189–1.590
Sex												
Female	Ref	–	–	Ref	–	–	Ref	–	–	Ref	–	–
Male	1.067	0.159	0.975–1.169	1.048	0.312	0.957–1.149	1.054	0.264	0.961–1.157	1.051	0.284	0.959–1.152
Marital status												
Married	Ref	–	–									
Single	0.942	0.622	0.743–1.194									
Divorced or widowed	1.078	0.083	0.990–1.173									
Living situation												
Not living alone				Ref	–	–						
Living alone				1.066	0.170	0.973–1.168						
Education												
Elementary school or junior high school							Ref	–	–			
High school or junior high school under the old system							0.963	0.506	0.861–1.077			
Vocational school, junior college, technical school, university, college, graduate school, or other							0.906	0.263	0.762–1.077			
Work status												
Working										Ref	–	–
Not working										1.053	0.215	0.970–1.143
	**Model 5**			**Model 6**			**Model 7**			**Model 8**		
	**aPR**	**P-value**	**95% CI**	**aPR**	**P-value**	**95% CI**	**aPR**	**P-value**	**95% CI**	**aPR**	**P-value**	**95% CI**
Age												
20–59 years	Ref	–	–	Ref	–	–	Ref	–	–	Ref	–	–
60–79 years	1.274	< 0.001	1.115–1.456	1.283	< 0.001	1.123–1.466	1.257	0.001	1.098–1.439	1.295	< 0.001	1.130–1.484
80 years or older	1.404	< 0.001	1.220–1.616	1.409	< 0.001	1.227–1.619	1.375	< 0.001	1.192–1.587	1.455	< 0.001	1.252–1.692
Sex												
Female	Ref	–	–	Ref	–	–	Ref	–	–	Ref	–	–
Male	1.054	0.265	0.961–1.155	1.056	0.234	0.965–1.156	1.061	0.202	0.968–1.163	1.007	0.902	0.906–1.119
Public assistance												
Not receiving	Ref	–	–									
Receiving	0.905	0.546	0.654–1.252									
Physical activity												
Exercising				Ref	–	–						
Not exercising				1.092	0.199	0.955–1.248						
Smoking status												
Ex-smoker or never smoker							Ref	–	–			
Current smoker							0.929	0.408	0.779–1.107			
Alcohol intake												
Not having hazardous drinking or more										Ref	–	–
Having hazardous drinking or more										1.105	0.118	0.975–1.253

*aPR* adjusted prevalence ratio, *CI* Confidence interval

Total number of participants = 355; number of participants with multimorbidity = 301

Explanatory variables: age, sex, and marital status in Model 1; age, sex, and living situation in Model 2; age, sex, and education in Model 3; age, sex, and work status in Model 4; age, sex, and public assistance in Model 5; age, sex, and physical activity in Model 6; age, sex, and smoking status in Model 7; and age, sex, and alcohol intake in Model 8

The results of Poisson regression with robust error variance, with complex multimorbidity as an objective variable, are shown Table 4. Age 60 years or older (compared with age 20–59 years) was found to be associated with complex multimorbidity (adjusted prevalence ratio [aPR] = 1.887 and 1.831; 95% confidence interval [CI] = 1.304–2.731 and 1.189–2.820 for ages 60–79 years and 80 years or older, respectively). Being unemployed was also associated with complex multimorbidity (aPR = 1.251; 95% CI = 1.031–1.518). However, having hazardous drinking or more was inversely associated with complex multimorbidity (aPR = 0.745; 95% CI = 0.556–0.999).

**Table 4:** Poisson regression with robust error variance, with complex multimorbidity as an objective variable, and adjusted prevalence ratios

	aPR	*P*-value	95% CI
Age			
20–59 years	Ref	–	–
60–79 years	1.887	0.001	1.304–2.731
80 years or older	1.831	0.006	1.189–2.820
Sex			
Female	Ref	–	–
Male	1.089	0.413	0.887–1.338
Marital status			
Married	Ref	–	–
Single	0.618	0.216	0.288–1.325
Divorced or widowed	1.198	0.119	0.955–1.502
Living situation			
Not living alone	Ref	–	–
Living alone	1.136	0.306	0.890–1.449
Education			
Elementary school or junior high school	Ref	–	–
High school or junior high school under the old system	0.803	0.101	0.618–1.044
Vocational school, junior college, technical school, university, college, graduate school, or other	0.819	0.277	0.571–1.174
Work status			
Working	Ref	–	–
Not working	1.251	0.023	1.031–1.518
Public assistance			
Not receiving	Ref	–	–
Receiving	0.630	0.077	0.378–1.051
Physical activity			
Exercising	Ref	–	–
Not exercising	1.152	0.289	0.887–1.497
Smoking status			
Ex-smoker or never smoker	Ref	–	–
Current smoker	1.043	0.796	0.757–1.438
Alcohol intake			
Not having hazardous drinking or more	Ref	–	–
Having hazardous drinking or more	0.745	0.049	0.556–0.999

*aPR* adjusted prevalence ratio, *CI* Confidence interval

Total number of participants = 355; number of participants with complex multimorbidity = 191

## Discussion

The population-based prevalence of multimorbidity on a remote island in Japan was consistent with previous reports in the country whereas that of complex multimorbidity was not. The multimorbidity prevalence by age group had an upward trend with increasing age. Multimorbidity was associated with age 60 years or older. Additionally, complex multimorbidity was associated with age 60 years or older, being unemployed, and not having hazardous drinking or more.

Our study findings regarding the population-based prevalence of multimorbidity among those aged ≥ 20 on a remote island in Japan were consistent with those reported previously [11, 16]. Using self-reported data collected in a nationwide cross-sectional survey, Aoki et al. reported that the prevalence among individuals aged 16–84 years was 29.9% and the prevalence among individuals aged 65–84 years was 62.8% [11]. Kato et al. reported that the prevalence among individuals aged 65 years or more was 52.0%, using self-reported data collected in a nationwide cohort study [16]. In this study, we used data obtained directly from medical records, which can be expected to be more accurate but was restricted to a remote island area in Japan, as compared with nationwide data. Each of these studies has its own strengths and limitations, but similar results among these studies mutually support the reliability of the findings of each study. Additionally, the findings were consistent with the population-based prevalence of multimorbidity reported in systematic reviews from other countries [3, 8, 40].

The upward trend of multimorbidity prevalence by age group was also consistent with that reported in other countries [2, 8]. However, the prevalence in this study, especially that in the age group 80 years or older, tended to be slightly lower compared with that in other countries, as reported in systematic reviews, with prevalence ranges approximately 15%–50% at age 40 years, 40%–75% at age 60 years, and 70%–85% at age 80 years [2, 8]. This may be owing to underestimation of the prevalence of multimorbidity within the medical context of Tarama Island. Patients who are highly dependent on medical and nursing services, such as patients with terminal cancer, or those who are highly dependent on advanced medical services, such as those receiving dialysis, would be forced to move off the island because of a lack of medical and nursing resources. These patients are more likely to have multimorbidity; thus, their exclusion in this study could have led to underestimation of the prevalence of multimorbidity.

The population-based prevalence of complex multimorbidity in this study differed from that of previous studies in Japan. In particular, Kato et al. reported a 19.5% prevalence of complex multimorbidity among individuals aged ≥ 65 years, which is substantially lower than the prevalence in our study [16]. Kato et al. included older Japanese adults who were functionally independent (defined as not receiving public long-term care insurance) and not receiving any nursing care or home care, and they counted the number of body systems affected using only 17 diseases. This might have resulted in underestimation of the prevalence of complex multimorbidity in this age group. In population-based studies using medical records, all disease data for both functionally independent and dependent patients can be collected. To estimate the prevalence of complex multimorbidity, comprehensive data collection may be necessary. Similarly, compared with reports from other countries, prevalence varies substantially across studies owing to wide variation in factors, such as the number of study participants and their characteristics as well as the method used to count the number of morbidities [41–43]. Further studies, such as meta-analyses, may be warranted to validate the prevalence of complex multimorbidity.

We identified several factors that were associated with multimorbidity or complex multimorbidity. Age is the most well-established risk factor for multimorbidity [8]. A systematic review of the determinants of multimorbidity in primary care reported a significant positive association between age and the prevalence of multimorbidity [8]. In Japan, Mitsutake et al. reported age to be determinants of multimorbidity [13]. Similar results were obtained in this study regarding not only multimorbidity but also complex multimorbidity. Given that ageing is a main risk factor for developing a variety of diseases [44], this result is not surprising. Additionally, being unemployed was found to be related to complex multimorbidity. A few studies have reported an inverse association between the prevalence of multimorbidity and socioeconomic status [8]. During the past two decades, social determinants of health (SDH) have been drawing increasing attention, and unemployment is one of the key risk factors for poor health outcomes and premature death [45]. In contrast, our findings showed that having hazardous drinking or more was less likely to be associated with complex multimorbidity. Considering the fact that alcohol consumption has been identified to be a causal factor for more than 200 diseases, including both physical and psychiatric conditions [46, 47], we may need to consider reverse causality. Therefore, it may be reasonable to assume that individuals with complex multimorbidity abstain from consuming the amount of alcohol defined as indicating hazardous drinking or more because of their health problems or medications they are taking [48]. However, it should be noted that the upper bound of the aPR CI between having hazardous drinking or more and complex multimorbidity was nearly 1.00. To clarify the association, further research that includes a larger sample size or compares the association between different settings is needed. We did not find an association between being unemployed or not having hazardous drinking or more and multimorbidity; this may be because multivariate analysis regarding multimorbidity did not include all covariates that were included in multivariate analysis for complex multimorbidity, owing to a low number of events [39].

In this study, being unemployed as well as age was identified as being associated with complex multimorbidity, which indicated a possible association between the coexistence of diseases and SDH in Japan. In light of the influence exerted by SDH on health outcomes [45], conditions other than being unemployed may be associated with multimorbidity and complex multimorbidity. If so, non-medical approaches, such as social prescribing [49], will need to be promoted to provide better care for patients with multimorbidity and complex multimorbidity. More research that takes SDH into account is warranted, and policymaking based on evidence is essential for Japan. The present study can serve as a foundation for accumulating such evidence.

This study had several limitations. First, it was conducted at a single medical institution on a remote island in Japan. Therefore, the generalizability of the findings is limited. However, considering that patients in Japan are allowed to visit any medical institutions that they wish, it is difficult to obtain accurate data to conduct population-based studies in the country, other than in this type of study setting where geographical constraints prevent people from willingly attending other medical institutions located off the island. Second, although patients were consecutively included in our study, we excluded some patients who had no capacity to give consent. The main reason for this was dementia. Considering that patients with dementia are generally older adults and age 60 years or older was associated with multimorbidity/complex multimorbidity, failure in sampling could have led to underestimation of the prevalence of multimorbidity or complex multimorbidity. Third, for comparison purposes, multimorbidity was defined as having ≥ 2 among 17 chronic health conditions, in accordance with previous studies conducted in Japan. However, these 17 chronic health conditions may not satisfy the necessary types and sufficient number of conditions to measure multimorbidity. Considerable variation in how multimorbidity is measured has been reported [50]; thus, heterogeneity in methodological criteria results in variation in the multimorbidity prevalence [15]. Future studies should use the necessary types and sufficient number of conditions, according to consensus.

## Conclusions

The population-based prevalence of multimorbidity and its upward trend with increasing age on a remote island in Japan was consistent with previous reports in the country. Multimorbidity was associated with age 60 years or older, and complex multimorbidity was associated with age 60 years or older, being unemployed, and not having hazardous drinking or more. Our study findings indicated a possible association between the coexistence of diseases and SDH in Japan. To improve care for patients with multimorbidity and complex multimorbidity, more research that takes SDH into account is warranted, and evidence-based policymaking is essential for Japan. The present study can provide a foundation for accumulating such evidence.

## Data Availability

The datasets generated and/or analyzed during the current study are not available because consent for sharing of raw data was not obtained and the dataset could theoretically pose a threat to confidentiality.

## Abbreviations

STROBE: Strengthening the Reporting of Observational Studies in Epidemiology
JPY: Japanese yen
ICPC-2: International Classification of Primary Care, 2nd edition
AUDIT: Alcohol Use Disorders Identification Test
SD: Standard deviation
aPR: adjusted prevalence ratio
CI: Confidence interval
SDH: Social determinants of health
